# Factors associated with professionalism among ophthalmology medical residents

**DOI:** 10.5935/0004-2749.2024-0381

**Published:** 2025-09-10

**Authors:** Marcus Vinicius Cardoso de Souza, Alexandre Sampaio Moura

**Affiliations:** 1 Faculdade Santa Casa Belo Horizonte, Belo Horizonte, MG, Brazil

**Keywords:** Internship and residency, Ophthalmology, Pro fessional competence, Education, professional, Physician-patient relations, Surveys and questionnaires

## Abstract

**Purpose:**

This study aimed to examine factors related to the professionalism of ophthalmology residents.

**Methods:**

A cross-sectional study was carried out involving 48 ophthalmology residents in Brazil. Professionalism was assessed using the professionalism mini-evaluation exercise, completed by both preceptors and residents, and the Pennsylvania State College of Medicine Professionalism Questionnaire, completed by the residents. The association between the professionalism score assigned by the preceptor through the professionalism mini-evaluation exercise and various sociodemographic and educational variables was assessed. The correlation between the residents’ self-assessment across both instruments and the preceptor’s assessments was measured using Spearman’s Rho.

**Results:**

All 48 residents were included, with equal representation across the 3 years of residency. The majority were female (58.3%) and between 25 and 29 years old (66.7%). The average professionalism score on the professionalism mini-evaluation exercise given by the preceptors was 3.0 (75%). A significant association was found between the year of training and the score in the doctor-patient relationship domain, with first-year residents showing lower scores (p=0.002). Male residents had higher scores in the “Interprofessional” domain (p=0.031). Graduates from private medical schools scored higher in both the “doctor-patient relationship” (p=0.015) and “reflective skills” (p=0.033) domains. Lower interest in professionalism was linked to lower scores in the “Interprofessional relationships” (p=0.033) and “time management” (p=0.003) domains. A strong correlation was observed between preceptor’s professionalism mini-evaluation exercise scores and residents’ self-assessed professionalism mini-evaluation exercise scores (r=0.917). However, the correlation between the self-assessed professionalism mini-evaluation exercise and the Pennsylvania questionnaire scores was weak (r=0.226).

**Conclusion:**

Professionalism scores among ophthalmology residents were associated with year of training, gender, type of undergraduate education, and level of interest in the topic.

## INTRODUCTION

Medical professionalism encompasses the qualities, behaviors, and values that guide healthcare professionals in fulfilling their ethical duties toward patients, colleagues, and society. It involves a dedication to delivering high-quality care, professional competence and upholding ethical standards in medical practice^(1)^.

The American Medical Association understands that defines professionalism as treating all patients with dignity, respect, and empathy, regardless of their background or circumstances^(2)^. Similarly, the Royal College of Physicians and Surgeons of Canada characterizes medical professionalism as a sincere and deep commitment to the health and welfare of individual patients and society, demonstrated through ethical conduct, high personal behavioral standards, accountability to both the profession and society, physician-led regulation, and attention to one’s own health^(3)^. In general, different viewpoints on medical professionalism converge in emphasizing the importance of trust, the prioritization of patient welfare, and adherence to strong ethical principles in medical practice.

While professionalism is addressed in undergraduate medical education, applying theoretical concepts in clinical practice remains challenging^(4)^. Residency training appears to play a key role in cultivating professionalism among early-career physicians^(5)^. Medical residents often encounter situations that require the application of ethical principles and professional conduct. Nonetheless, a structured culture promoting reflective practice and the ongoing enhancement of professionalism is not widely established^(6)^.

The use of appropriate assessment tools is essential to support the development of professionalism within medical education. A review of 74 instruments designed to assess professionalism among healthcare professionals showed that these tools are applied in various ways^(7)^. Some studies have examined how preceptors use these instruments to directly assess students or residents during interactions with real or simulated patients, whereas others have explored their use by students or residents for self-evaluation. Two instruments frequently employed to assess professionalism in medical residents are the Professionalism Mini-Evaluation Exercise (P-MEX) and the Pennsylvania State College of Medici ne-Professionalism Questionnaire (PSCOM-PQ)^(8^,^9)^.

The P-MEX consists of 21 items distributed across four domains: doctor-patient relationship^(6)^, reflective skills^(6)^, interpersonal relationships^(6)^, and time management^(3)^. Each item is rated by an observer or self-assessed by the resident using a 4-point Likert scale^(8)^. The PSCOM-PQ includes 36 items equally divided among six domains: accountability, altruism, duty, enrichment, honesty and integrity, and respect. Each item in the PSCOM-PQ presents a statement that residents evaluate through self-assessment using a 5-point Likert scale. Furthermore, residents rated the relevance of each statement to their clinical practice. Both instruments have been translated and culturally adapted for use in Brazilian Portuguese^(10^-^12)^.

Although suitable instruments are available, professionalism is infrequently assessed among medical residents during routine practice^(13)^. Despite being a fundamental component of medical training, there is a prevailing assumption that residents have already acquired professionalism by the time they complete medical school^(14)^. Evaluating how professionalism evolves throughout residency and identifying factors that support its development may contribute to the creation of effective educational strategies. This study aimed to investigate factors associated with the development of professionalism in ophthalmology residents.

## METHODS

### Study design

This was a cross-sectional observational study conducted between August 2022 and March 2023. The study was approved by the ethics committee of *Santa Casa de Belo Horizonte* (CAAE 61311622.8.0000.5138), and all participants provided written informed consent.

### Setting

The study was carried out at *Hospital da Santa Casa de Belo Horizonte*, located in Belo Horizonte, Brazil, which offers a 3-year residency program in Ophthalmology. This program is accredited by both the *Conselho Brasileiro de Oftalmologia* and the National Commission of Medical Residency (CNRM).

### Population

All residents enrolled in the ophthalmology training program at *Hospital da Santa Casa de Belo Horizonte* were invited to participate in the study. In addition, ophthalmology residents from another public institution (*Hospital Evangélico*) who were conducting their activities at *Hospital da Santa Casa* during the study period were also invited. Residents who were on sick leave or on vacation or who did not complete the study questionnaires were excluded. A briefing session was held to explain the study’s objectives and procedures to the residents. Informed consent was obtained from all participants, who were informed that participation was entirely voluntary. The scores obtained during the study were kept confidential and were not disclosed to the program director.

### Procedures

Throughout the study, a single preceptor directly observed all participants using the P-MEX instrument. The P-MEX consists of 21 items distributed across four domains. Each item is rated on a 4-point Likert-type scale. The overall P-MEX score is calculated as the mean of all item scores, ranging from 1 to 4^(10)^. The domain scores vary according to the number of items: doctor-patient relationship skills (6 items, 24 points), reflective skills (6 items, 24 points), time management (3 items, 12 points), and interprofessional relationship skills (6 items, 24 points).

Observations were carried out in two settings: ambulatory care and emergency care. A total of 384 medical encounters were evaluated, with 4 encounters per resident in each setting. These assessments were integrated into the preceptor’s regular duties, and residents were unaware of when they would be observed. At the end of the rotation period, residents completed a questionnaire before receiving feedback from the preceptor. The questionnaire administered to the residents included three parts: the first part collected sociodemographic and academic information; the second part included the P-MEX items; and the third part comprised the PSCOM-PQ items. The PSCOM-PQ is a 36-item self-assessment tool organized into six domains: accountability, enrichment, honor and integrity, altruism, duty, and respect. Responses are recorded on a 5-point Likert scale. The PSCOM-PQ total score is calculated either as the mean score (ranging from 1 to 5) or as a sum score (ranging from 36 to 180)^(11^,^12)^. Each domain, containing six items, has a score range from 6 to 30.

### Data analysis

Participants’ characteristics were summarized using means and standard deviations for quantitative variables and frequencies for qualitative variables. The relationship between professionalism scores and participants’ characteristics was assessed using the Kruskal-Wallis test. Differences between self-assessed P-MEX scores and those assigned by the preceptor were examined with the Wilcoxon signed-rank test. Correlations between scores from the two professionalism instruments (P-MEX and PSCOM-PQ) were assessed using Spearman’s Rho, a nonparametric test. Statistical analyses were conducted using IBM SPSS Statistics (version 29). A p-value ≤0.05 was considered statistically significant.

## RESULTS

Of the 50 eligible residents, 48 agreed to participate. Women slightly outnumbered men, comprising 58.3% of the sample, and the majority of participants were aged between 25 and 29 years. The participants were evenly distributed across the 3 years of training, with 16 residents in each year. Most had graduated from private universities (Table 1). Forty-four residents were from *Hospital da Santa Casa*, and four were from *Hospital Evangélico*. There were no dropouts during the study.

**Table 1:** Characteristics of the participants (n=48)

	N (%)
**Year of training**	
R1	16 (33.3)
R2	16 (33.3)
R3	16 (33.3)
**Gender**	
Male	20 (41.6)
Female	28 (58.3)
**Age group (years)**	
20–24	2 (4.1)
25–29	32 (66.6)
30–34	14 (29.1)
**Undergraduate medical education**	
Private	34 (70.8)
Public	14 (29.1)
**Interest in religion**	
None	4 (8.3)
Small	13 (27.0)
Moderate	15 (31.2)
Large	16 (33.3)
**Interest in philosophy**	
None	18 (37.5)
Small	21 (43.7)
Moderate	7 (14.5)
Large	2 (4,1)
**Interest in the study of professionalism**	
None	3 (6.2)
Small	4 (8.3)
Moderate	24 (50.0)
Large	17 (35.4)
**Was professionalism addressed during medical school?**	
No	10 (20.8)
Yes, integrated with other academic disciplines	29 (60.4)
Yes, in a specific academic discipline	9 (18.7)
**Is professionalism addressed during residency?**	
No	16 (33.3)
Yes, integrated with other academic disciplines	32 (66.6)
Yes, in a specific academic discipline	0 (0.0)
**Do you think discussing professionalism during residency is important/relevant?**	
No	20 (41.6)
Yes	28 (58.3)

Forty-one residents (85.4%) considered professionalism to be essential in medical practice; however, 44.6% did not believe it should be a focus during residency training. One-third of the residents reported not recalling any discussions about professionalism during their residency. Psychological frailty—defined as a moderate to high need for psycho-pedagogical support-was present in 83.3% of the participants (Table 1).

### Factors associated with professionalism scores in the P-MEX

**Figure 1:**
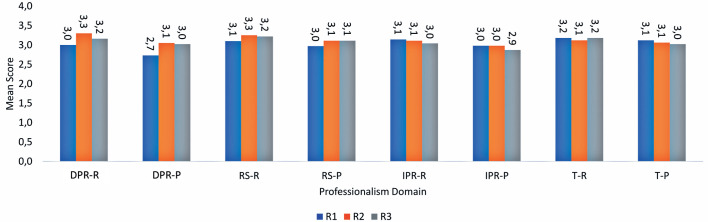
Average P-MEX scores as self-assessed by residents and assigned by the preceptor, shown by domain and training year. DPR-R= doctor-patient relationship - resident self-assessment; DPR-P= doctor-patient relationship - preceptor’s assessment; RS-R= reflective skills - resident self-assessment; RS-P= reflective skills - preceptor’s assessment; IPR-R= interprofessional relationship - resident self-assessment; IPR-P= interprofessional relationship - preceptor’s assessment; T-R= time management - resident self-assessment; T-P= time management - preceptor’s assessment.

Figure 1 presents the professionalism scores assigned by the preceptor and self-assessed by the residents across each P-MEX domain. The overall mean professionalism score given by the preceptor was 3.0 out of 4.0 (75.0%), while the residents’ self-assessed mean score was 3.2 out of 4.0 (78.4%). A strong positive correlation was found between the preceptor’s scores and residents’ self-assessments (Spearman’s Rho=0.917; p<0.001), although the two sets of scores differed significantly (p<0.001).

No association was identified between the year of training (R1, R2, and R3) and the overall P-MEX scores assigned by the preceptor (Table 2). However, there was a significant association between the year of training and scores in the “Doctor-Patient Relationship” domain, with higher scores for R2 and R3 compared to R1 (18.0 vs. 16.0; p=0.002).

**Table 2:** Median P-MEX scores according to residents’ characteristics

Variable	Doctor-Patient Relationship	Reflective Skills	Interprofessional Relationship	Time Management	Global
**Year of Training**					
R1	16.0**	18.0	17.5	09.0	61.5
R2	18.0**	19.0	18.0	09.0	63.0
R3	18.0**	18.0	17.0	09.0	63.0
**Gender**					
Male	18.0	18.0	18.0*	09.0	63.0
Female	17.5	19.0	17.0*	09.0	63.0
**Undergraduate training**					
Private	18.0*	19.0*	17.0	09.0	63.0
Public	16.5*	18.0*	17.0	09.0	63.0
**Interest in professionalism**					
Yes	18.0	19.0	18.0	10.0**	63.5
No	18.0	18.0*	17.0*	09.0*	62.0

Kruskal-Wallis test for independent samples.

* p<0.05,

** p<0.01; Fonte: Dados da pesquisa (2023).

A difference was also found in the “Interprofessional Relationship” domain between male and female residents, with men scoring higher than women (18.0 vs. 17.0; p=0.031).

Regarding undergraduate medical education, residents who graduated from private institutions scored higher in both the “Doctor-Patient Relationship” domain (p=0.015) and the “Reflective Skills” domain (p=0.033) compared to those from public schools.

Residents with greater interest in studying professionalism had higher scores in the “Interprofessional Relationship” and “Time Management” domains than those with less interest (p=0.033 and p=0.003, respectively).

### Factors associated with self-assessed professionalism scores in the PSCOM-PQ

**Figure 2:**
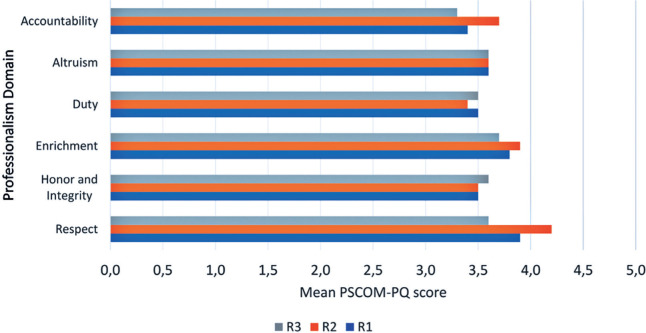
Average PSCOM-PQ scores self-assessed by residents, stratified by domain and training year.

Figure 2 shows the self-assessed scores by residents for each domain of the PSCOM-PQ. The average overall score on the PSCOM-PQ was 3.6 out of 5.0 (72.2%). Among first-year residents (R1), the highest score was for the “enrichment” domain, closely followed by “respect”. For residents in the second and third years (R2 and R3), the highest scores were in the “respect” domain. The lowest scoring items were “accountability” for R1 and R3 and “duty” for R2. No statistically significant differences were found in PSCOM-PQ scores across the six domains among the different years of training (Table 3). Additionally, PSCOM-PQ scores showed no association with gender, undergraduate medical education background, or level of interest in professionalism.

**Table 3:** Analysis of the relationship between self-rated PSCOM-PQ scores and residency year

Domain	R1	R2	R3	Total	p-value
Accountability	20.5	22.0	21.0	22.0	0.198
Altruism	20.5	22.0	21.0	21.0	0.683
Duty	21.0	21.0	21.0	21.0	0.707
Enrichment	22.5	22.5	23.0	23.0	0.382
Honor and Integrity	21.0	22.0	20.0	20.5	0.540
Respect	22.0	23.0	23.0	23.0	0.574
Total	127.5	132.5	129.0	130.5	0.524

R1= first year of training; R2= second year of training; R3= third year of training.

### Analysis of the relevance ranking of the different PSCOM-PQ items

**Figure 3:**
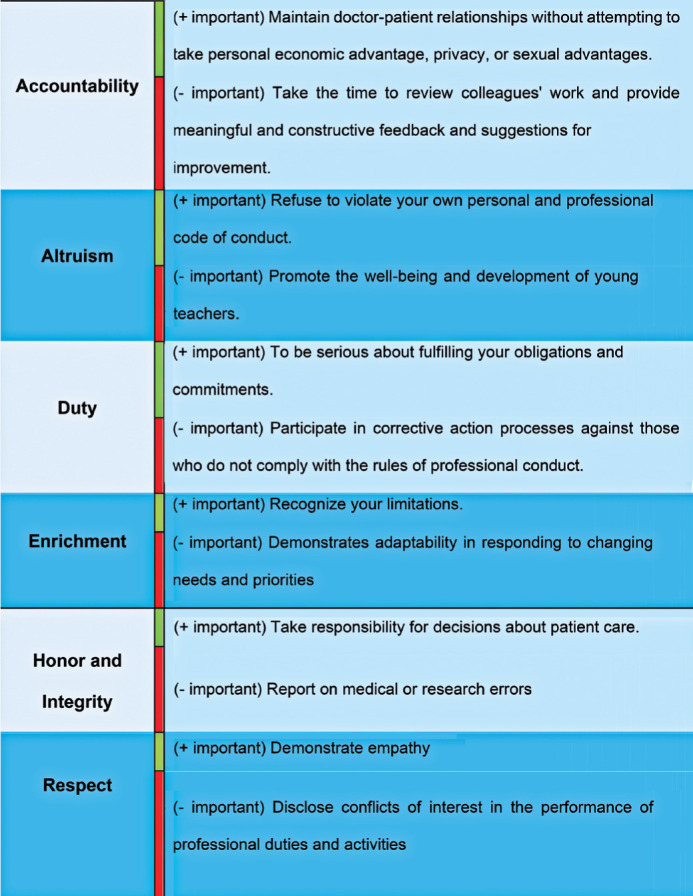
Grouping of the relevance ratings of PSCOM-PQ questionnaire items.

Figure 3 displays the ranking of how residents perceived the relevance of each PSCOM-PQ statement. The items considered most relevant were those related to the physician’s individuality and the doctor-patient relationship. Conversely, institutional aspects such as respecting and adhering to workplace rules (duty and respect) were among the items ranked as less relevant by the residents.

### Correlation between the PSCOM-PQ and P-MEX questionnaires

Although both questionnaires are designed to assess professionalism, the overall correlation between the P-MEX and PSCOM-PQ scores was weak (r=0.226).

## DISCUSSION

Using two different instruments, we found high professionalism scores among ophthalmology residents. Our findings are comparable to those from a study in Japan involving residents and healthcare professionals, which reported a P-MEX mean score of 3.25, and a Finnish study with psychiatry residents, where the mean score was 3.26^(15^,^16)^. However, in the Finnish study, residents’ self-assessed scores were slightly lower than those given by preceptors (3.01 vs. 3.26). In contrast, our study showed higher self-assessment scores by residents compared to the preceptor’s ratings, which may reflect cultural differences. Despite these score differences, there was a strong correlation between preceptor-assigned and resident self-assessed P-MEX scores. This is important because if residents’ self-evaluations of professionalism corresponds closely with external observations, direct observation may be less necessary, potentially saving time and resources.

We also aimed to identify factors linked to the deve lopment of professionalism among ophthalmology residents. Unexpectedly, there was no statistically significant difference in the overall P-MEX scores between residents at different training levels. The consistently high scores from the first year suggest that residents began their training with well-developed professional skills and maintained this level throughout their residency. These findings are consistent with a study involving residents from various specialties at a large tertiary teaching hospital. That study, which used a different professionalism measure—the Learners Attitude of Medical Professionalism Scale (LAMPS)—found no difference in self-reported attitudes between senior and junior residents^(17)^. The authors proposed two explanations that may also apply here: (i) the instrument may have lacked sensitivity to detect subtle changes in professionalism, preventing differentiation among residents, or (ii) a ceiling effect, where residents already scoring highly at the start of residency have limited room for measurable improvement during training.

Although no significant change was observed in the overall professionalism scores throughout training, there was a statistically significant improvement in the “Doctor-Patient Relationship” domain scores. Clinical experience appears to enhance skills such as actively listening to patients, demonstrating interest and respect, recognizing and addressing patient needs, and advocating on their behalf, all of which are crucial to this relationship^(18^,^19)^.

In terms of gender differences, male residents scored higher in the “Interprofessional Relationships” domain of the P-MEX. This domain refers to the physician’s interactions with other healthcare personnel, including colleagues, professionals, and administrators. Bell et al. suggested that interprofessional collaboration in healthcare may be impeded by status differences between men and women and how these inequalities are “exercised and perpetuated within healthcare delivery”^(20)^.

When comparing P-MEX scores between residents who graduated from public and private colleges, higher scores were observed in the “Doctor-Patient Relationship” and “Reflective Skills” domains among those who completed private undergraduate medical education. Most private medical schools in Brazil were established following the National Curriculum Guidelines of 2003 and 2014. These guidelines recommend incorporating active teaching and learning methods, promoting early student integration with the public health system, and aiming to train physicians with critical and reflective thinking^(21)^. Although public schools are also required to follow these guidelines, their medical curricula tend to be older, and changes are implemented more gradually. Our findings may reflect the positive impact of these newer curriculum guidelines, which likely benefit students from private medical schools.

High professionalism scores were also reported when residents self-assessed using the PSCOM-PQ. Our findings were similar to a study involving Spanish residents, which found about 70% positive responses across all PSCOM-PQ domains^(22)^. In that study, altruism scored lowest, while respect scored highest. In contrast, our study showed the highest scores for respect and excellence, with honesty receiving the lowest scores. These differences may be influenced by cultural factors.

In our study, the PSCOM-PQ items rated as most rele vant were as follows: (i) maintaining doctor-patient relationships that avoid exploitation for personal financial gain, privacy, or sexual advantage, (ii) refusing to breach one’s personal and professional code of conduct, (iii) fulfilling commitments and obligations conscientiously, and (iv) acknowledging one’s own limitations. These results highlight a focus on the patient, responsibility, and professional humility.

The PSCOM-PQ items considered less relevant by the residents included (i) taking time to review colleagues’ work and offering meaningful, constructive feedback for improvement, (ii) supporting the welfare and development of junior faculty, (iii) participating in corrective actions against those who fail to meet professional conduct standards, and (iv) showing adaptability in res ponse to changing needs and priorities.

Our findings may reflect traits commonly associated with the so-called Generation Y or Millennials, who make up most of the residents in this study. Individuals from Generation Y are characterized by extensive use of technology, highly involved parents, lower levels of self-reliance, and limited coping skills. Key workplace values for this generation include online social connectedness, teamwork, freedom of speech, close relationships with authority figures (similar to those with their parents), creativity, and a healthy work-life balance^(23)^.

A significant proportion of our participants reported a moderate to high need for psycho-pedagogical support. A study involving undergraduate and postgraduate medical trainees in Canada found that feeling psychologically supported decreases the risk of psychological distress and burnout^(24)^. Maintaining a good work-life balance, job support, and control over work are important factors in reducing stress-related issues^(25)^.

Millennials possess distinct traits that influence professionalism, and preceptors need to recognize that generational differences call for new teaching methods. When instructing Millennials in professionalism, preceptors should encourage intentional training to enhance their emotional intelligence, which involves the ability to observe, understand, manage, and respond to both their own and others’ emotions^(26)^.

The fact that nearly half of the participants did not view studying professionalism as important is concerning and may reflect that professionalism education has traditionally been part of the “hidden curriculum” in medical training. A recent review emphasized that professionalism is a core competency and should be an integral part of surgical training. The authors recommend introducing more formal training to develop this competence, with evaluations starting early in residency and accompanied by proper feedback^(27)^.

 The PSCOM-PQ domain scores were not linked to the sociodemographic or educational variables analyzed and showed only a weak correlation with the P-MEX scores. This weak correlation may be due to the different approaches of the two instruments: while the P-MEX evaluates directly observable behaviors like the doctor-patient relationship, reflective skills, time management, and interprofessional relationships, the PSCOM-PQ includes more conceptual items that assess learners’ attitudes toward professionalism domains such as accountability, enrichment, honor and integrity, altruism, duty, and respect. Therefore, our findings indicate that these tools can be used complementarily to assess medical trainees.

This study has some limitations. It included residents from only two institutions, which may restrict the generalizability of the results to other educational environments, and it did not include comparisons with other clinical or surgical specialties. However, one study suggests no difference in professionalism scores between surgical and nonsurgical specialties, and another found similar professionalism scores between ophthalmology and pediatric residents^(27^,^28)^. Another limitation concerns the assessment of residents by a single preceptor. Due to resource constraints, only one assessor was used, which may have introduced observer bias. Future research involving multiple observers could help minimize this bias. A third limitation is the possibility that residents altered their performance because they knew they were being assessed. To mitigate this, the preceptor assessed residents during routine activities, and the residents were unaware of the exact timing of the evaluations.

In summary, ophthalmology residents showed high professionalism scores. Higher scores were found in the “Reflective Skills” and “Time Management” domains, while lower scores were observed in the “Doctor-Patient Relationship” domain. Factors associated with P-MEX domains included year of training, gender, and type of undergraduate medical education.

## Data Availability

The datasets generated during and/or analyzed during the current study are available in the manuscript.
